# Prophylactic Treatment of Hepatitis C Virus Infection After Kidney Transplantation with the Combination of Glecaprevir/Pibrentasvir and Sofosbuvir in a Highly Sensitized Hepatitis C Virus-Negative Recipient: A Case Report and Review of the Literature

**DOI:** 10.3390/biomedicines13020472

**Published:** 2025-02-14

**Authors:** Tanja Belčič Mikič, Igor Sterle, Mojca Matičič, Miha Arnol

**Affiliations:** 1 Department of Nephrology, University Medical Centre Ljubljana, 1000 Ljubljana, Slovenia; miha.arnol@kclj.si; 2 Faculty of Medicine, University of Ljubljana, 1000 Ljubljana, Slovenia; mojca.maticic@kclj.si; 3 Department of Urology, University Medical Centre Ljubljana, 1000 Ljubljana, Slovenia; igor.sterle@kclj.si; 4 Clinic for Infectious Diseases and Febrile Illnesses, University Medical Centre Ljubljana, 1000 Ljubljana, Slovenia

**Keywords:** kidney transplantation, HCV RNA, direct-acting antiviral (DAA), glecaprevir/pibrentasvir, sensitization, case report

## Abstract

**Background**: Since the discovery of successful direct-acting antiviral (DAA) treatment, kidneys from hepatitis C virus (HCV) RNA-positive donors represent a new opportunity to expand the organ donor pool for HCV-negative recipients. **Case presentation**: In this paper, we describe a unique case of transplantation of an HCV genotype 3a-infected kidney into an HCV-negative recipient who was highly sensitized, with a virtual panel-reactive antibody level of 99.96%. Prior to the kidney transplantation, the recipient received DAA treatment with glecaprevir/pibrentasvir as a viable prophylactic strategy. Post-transplant, the recipient received a triple-combination DAA regimen with glecaprevir/pibrentasvir/sofosbuvir, which continued for 12 weeks. Subsequently, viral load was undetectable at 12 and 24 weeks after treatment, with no significant adverse events associated with DAA therapy. A 12-month post-transplantation biopsy revealed mixed rejection requiring treatment. The 19-month follow-up showed a favorable outcome regarding the function of the kidney allograft and the recipient’s quality of life. HCV-positive transplantation allowed our recipient to receive a kidney from an immunologically compatible donor without donor-specific antibodies and the need for desensitization strategies. **Conclusions**: Each transplant center should decide on the selection of candidates for kidney transplantation from HCV RNA-positive donors to HCV-negative recipients, the availability and choice of DAA treatment, and post-transplant follow-up. Our case emphasizes the need for early DAA treatment based on viral load and HCV genotyping, as well as for careful post-transplant surveillance including protocol biopsies.

## 1. Introduction

Highly sensitized patients with a virtual panel-reactive antibody (vPRA) level >85% have limited access to kidney transplantation because of a shortage of immunologically acceptable donors and exposure to the complications of long-term dialysis treatment [1,2]. Until recently, kidneys from deceased donors infected with hepatitis C virus (HCV) who are HCV RNA-positive were often discarded despite good organ quality or only used in HCV-positive recipients. With the discovery of successful direct-acting antiviral (DAA) treatment more than a decade ago [3,4] and reports of its effective use in kidney transplant recipients [5], transplantation from HCV RNA-positive donors to HCV RNA-negative recipients (HCV RNA D+/R−) suddenly became a new opportunity to expand the organ donor pool and shorten the waiting time. Nevertheless, the rate of discard of HCV-positive kidneys remained high even after the introduction of successful direct-acting antivirals (DAAs). A report from the Organ Procurement and Transplantation Network (OPTN) showed that in 2019, kidneys from HCV RNA-positive donors still had a 48% higher chance of being discarded compared to kidneys from aviremic, HCV-seronegative donors [6]. Reasons for not using HCV RNA-positive organs more frequently include insurance delays or refusal of DAA therapy in some countries, current local laws and regulations, risk of viral transmission, fear of potential complications, and lack of data on long-term follow-up [6,7].

In our paper, we present a unique case of HCV RNA D+/R− kidney transplantation to a highly sensitized recipient with 99.96% vPRA and provide a review of the current evidence on kidney transplantation from HCV RNA-positive kidney donors.

## 2. Detailed Case Description

### 2.1. The Donor

The donor was a 42-year-old woman with a body mass index of 42 kg/m^2^ who had a history of smoking (30 pack-years), moderate alcohol consumption, and various drug abuse including cocaine, heroin, crack, codeine, pregabalin, and amphetamines. She died of hypoxic brain injury due to asphyxia following an amphetamine overdose, and during this hospitalization, she was first diagnosed with active HCV infection. She had previously not received any treatment for HCV infection. The HCV RNA viral load was 4,780,000 IU/mL (6.68 log10 IU/mL). Sequence analysis of the HCV core fragment confirmed infection with HCV genotype 3a (Abbott Molecular, Des Plaines, IL, USA). Screening for hepatitis B, human immunodeficiency virus, cytomegalovirus (CMV), and *Treponema pallidum* infection was negative. Abdominal ultrasonography revealed a normal-sized liver with slightly hyperechogenic parenchyma and no suspicious lesions. Apart from cholecystolithiasis, there were no significant findings in the abdomen. Both kidneys were of normal size. The left kidney was 11 cm long with a 2.4 cm wide normal parenchyma and had no signs of obstruction. The right kidney was 11.5 cm long with a 2.5 cm wide normal parenchyma and had no signs of calculi or obstruction. Other tests performed were an electrocardiogram, a chest radiograph, a bronchoscopy, a heart ultrasound, a coronary angiography, and a breast ultrasound which revealed no abnormalities. Among the organs procured were the heart and both kidneys.

### 2.2. The Recipient

The kidney transplant recipient was a 31-year-old man with end-stage kidney failure of unknown etiology who started hemodialysis in 2008 at the age of 17. A kidney biopsy of his own kidneys was not performed. In April 2013, at the age of 22, he underwent a deceased-donor kidney transplantation in the right iliac fossa. Following the procedure, renal vein thrombosis developed which required a thrombectomy. The early post-transplant period was complicated by T cell-mediated rejection, which was treated with methylprednisolone and anti-thymocyte globulin. In March 2014, an indication biopsy due to deteriorating kidney function revealed mixed T cell-mediated and acute antibody-mediated rejection with positive donor-specific antibodies (DSAs), which eventually led to kidney transplant failure and removal of the kidney graft in October 2014. The patient restarted hemodialysis. He was re-enlisted on the Eurotransplant waiting list for the second transplantation in November 2020. However, he was highly sensitized with a vPRA level of 99.96% and finding a suitable donor within a reasonable timeframe seemed challenging.

**Figure 1:**
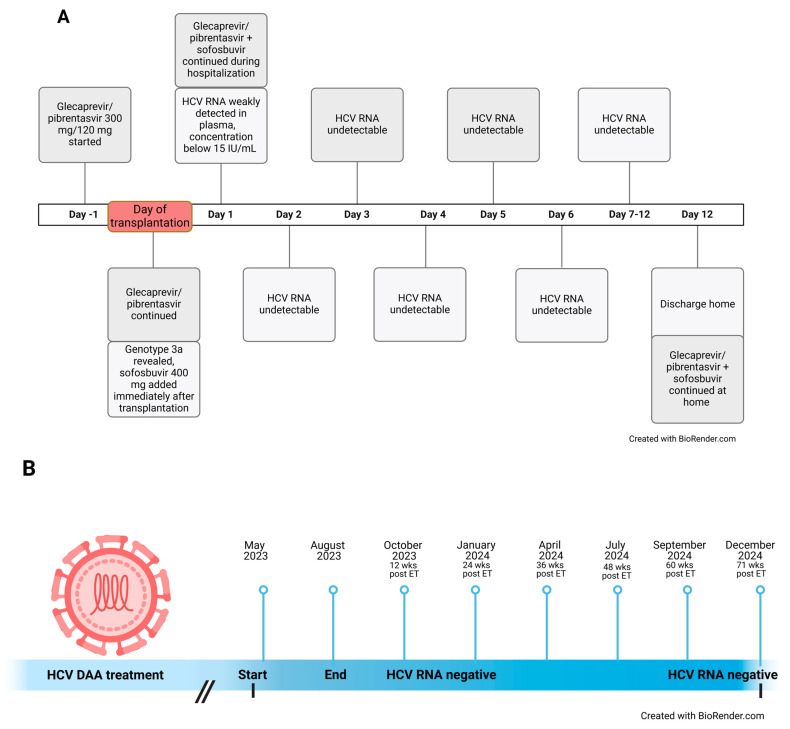
(**A**), Timeline of treatment and HCV RNA testing during hospitalization for kidney transplantation. (**B**) Outpatient follow-up examinations by an infectious disease specialist. HCV, hepatitis C virus; RNA, ribonucleic acid; DAA treatment, direct-acting antiviral treatment; wks, weeks; ET, end of treatment.

On 12 May 2023, he received a kidney offer from a 42-year-old donor infected with HCV. At that time, the HCV genotype was still unknown. Despite being highly sensitized, no DSAs were present at the time of transplantation, and human leukocyte antigen (HLA)-A, B, DR mismatch was 1, 0, 0. After being informed of the risks and benefits of transplantation with an HCV-infected kidney, including the need for DAA therapy, the patient provided informed consent for the procedure, which was performed the following day. One day prior to transplantation, the patient started prophylactic treatment with glecaprevir/pibrentasvir (G/P) 300 mg/120 mg. After HCV genotyping had been performed the next day and had revealed genotype 3a, sofosbuvir 400 mg was added to the daily therapy immediately after transplantation. Concomitantly, treatment was continued with G/P 300 mg/120 mg daily. The plasma HCV viral load was monitored daily after transplantation at first by using a quantitative polymerase chain reaction (PCR) test with the lower limit of detection defined at 15 IU/mL according to the manufacturer (COBAS 6800 System (Roche Molecular Systems, Branchburg, NJ, USA)). On the first day after transplantation, HCV RNA was detectable but unquantifiable. On the next and subsequent days, a qualitative PCR test was used with the lower limit of detection defined at 10 IU/mL according to the manufacturer (COBAS 6800 System (Roche Molecular Systems, Branchburg, NJ, USA)), yet the HCV RNA was undetectable. After discharge, the patient continued the same DAA treatment for 12 weeks and was regularly monitored by an infectious disease specialist who specializes in the treatment of patients with HCV infection. Liver function was monitored regularly and remained normal. The timeline of treatment and HCV RNA testing results during hospitalization for kidney transplantation is presented in Figure 1. 

Due to the high vPRA, the patient was treated with an immunosuppressive protocol for patients at high immunological risk (anti-thymocyte globulin induction and maintenance immunosuppression with methylprednisolone, mycophenolic acid, and tacrolimus). Kidney graft function after transplantation was immediate and serum creatinine (SCr) decreased to 136 µmol/L on the day of discharge 12 days after transplantation. The patient continued immunosuppression with tacrolimus, mycophenolic acid, and methylprednisolone. The SCr remained stable with values between 110 and 130 µmol/L. At the end of May 2023, anti-HLA-A11 DSAs with a mean fluorescence intensity (MFI) of 1890 were detected. No change in therapy was indicated and the DSAs were negative in the next test in August 2023.

In July 2023, two months after transplantation, polyoma BK viremia was detected (plasma BK virus concentration was 3030 IU/mL) and the patient was switched from mycophenolic acid to everolimus. In addition, the dose of tacrolimus was lowered to achieve lower plasma trough concentrations, and five doses of intravenous immunoglobulins (400 mg/kg in one dose) were administered. The introduction of everolimus resulted in the occurrence of neutropenia, which required treatment with filgrastim, a granulocyte-colony stimulating factor. Screening for neutropenia-inducing infections was negative for the presence of Epstein–Barr virus (EBV), human herpesvirus 6 (HHV-6), CMV, and parvovirus B19 in peripheral blood. Everolimus was discontinued and the patient was temporarily treated via dual immunosuppression with tacrolimus and methylprednisolone. Valganciclovir was switched to letermovir. All of the above measures led to the resolution of neutropenia. At the regular follow-up examinations with the infectious disease specialist, the HCV RNA plasma concentration was below the lower limit of detection of the qualitative PCR test and the liver function tests were within the normal range. The patient continued the prescribed DAA therapy for 12 weeks until 3 August 2023. The patient was in excellent condition with normal liver function and undetectable plasma HCV RNA concentration. Antiviral therapy was discontinued and the patient remained under close monitoring by an infectious disease specialist. In September 2023, the plasma concentration of polyoma BK virus decreased significantly and mycophenolic acid was reintroduced at a lower dose. The absence of HCV RNA detection was confirmed 12 weeks after the end of treatment (October 2023). The patient continued to be regularly examined by an infectious disease specialist every three months and the HCV RNA plasma concentration remained below the lower limit of detection of the qualitative PCR test.

In November 2023, six months after transplantation, kidney function suddenly deteriorated with an increase in SCr to 181 µmol/L. Ultrasound examination revealed urolithiasis in the implanted ureter from the donor kidney. A percutaneous nephrostomy was inserted and the calculi were later surgically removed. In the following months, kidney graft function was stable with SCr values between 125 and 150 µmol/L, proteinuria was mild (up to 430 mg/day), and the polyoma BK virus in plasma was undetectable. No new findings were detected during regular follow-up examinations at the Infectious Diseases Clinic and the patient was in excellent condition. In May 2024, the protocol biopsy of the transplanted kidney 1 year after transplantation showed signs of mixed chronic rejection (chronic active T cell-mediated and antibody-mediated rejection). Anti-HLA-A11 DSAs with a low MFI level (110) were again present in the peripheral blood. The patient was treated with pulse steroids and optimization of immunosuppression (increase in the dose of mycophenolic acid). He continued with triple immunosuppressive therapy. At the end of June 2024, his kidney function was stable (SCr was 131 µmol/L) with no significant proteinuria. No DSAs were present (all MFI levels were bellow 100) and no adverse events were reported during rejection treatment. At the last follow-up in December 2024, 19 months after transplantation, the patient was in excellent condition with no new clinical or laboratory findings. HCV RNA remained undetectable in plasma.

## 3. Discussion

Our case is a unique case of HCV RNA D+/R− kidney transplantation where the recipient was very highly sensitized with an almost 100% vPRA. Highly sensitized patients were commonly excluded from clinical trials investigating HCV RNA D+/R− kidney transplantation due to the increased risk of graft loss following rejection and the potential requirement for immunosuppressive therapy beyond standard of care including desensitization strategies pre-transplantation [8,9]. Studies investigating the type of DAA treatment, its duration, time of initiation, and its success in HCV RNA D+/R− kidney transplantation in the past years are presented in Table 1. Our case emphasizes that HCV RNA D+/R− kidney transplantation is feasible even in highly sensitized recipients with permanently negative HCV RNA and no HCV or treatment-related complications if prophylactic treatment is initiated.

Patients who receive kidneys from HCV-positive donors usually spend less time on the waiting list and receive kidneys from younger donors [10]. In our case, the patient had limited options for transplantation due to an almost 100% vPRA. He spent about three years on the waiting list for his second transplant. Receiving an immunologically suitable kidney allowed for successful transplantation while avoiding desensitization strategies that could increase the risk of infectious complications after transplantation [11]. Due to the higher immunologic risk with potential cellular memory, he received an immunosuppressive protocol with anti-thymocyte globulin and remained on triple immunosuppressive therapy (tacrolimus, mycophenolic acid, methylprednisolone) after transplantation. He also continued to take DAA.

According to the guidelines of the American Association for the Study of Liver Diseases-Infectious Diseases Society of America (AASLD-IDSA), DAA treatment in HCV RNA D+/R− kidney transplantation should be either prophylactic or pre-emptive and started before or at the latest in the first 7 days after transplantation. Treatment is recommended for 8 to 12 weeks [12]. Early treatment initiation shows an advantage in terms of virologic failure (relapse/non-response) [13]. 

Recently, two studies investigated the success of delayed treatment initiation (beyond 7 days post-transplant) [13,14], which could avoid DAA treatment in patients who do not develop viremia, enable targeted selection of antiviral medication based on concomitant diseases and prescribed medication, and does not require immediate insurance authorization. In the HCV-target retrospective–prospective observational multicenter study, non-liver solid organ HCV RNA D+/R− transplantations were carried out including 79 (50%) kidney transplantations [13]. Early treatment was initiated within 7 days post-transplant and was given from a median of 7 days to 12 weeks depending on the chosen drug combination, whereas the late-treatment regimen was initiated a median of 31 days post-transplant and was given for 12 weeks regardless of the chosen drug combination. The number of patients with quantifiable HCV RNA was significantly higher in the late DAA treatment initiation group. Sustained virologic response 12 weeks after termination of treatment (SVR12) rates were higher in the early DAA treatment initiation group although not significantly, with virologic failure (relapse/non-response) detected in five patients who started DAA treatment late (5/102 = 4.9%). Fibrosing cholestatic hepatitis that developed as a result of virologic failure was detected in one patient. Interestingly, two patients (2/102 = 1.96%) in the late DAA treatment initiation group did not develop HCV viremia and did not require DAA treatment. Unfortunately, different standards of care and treatment regimens between centers and missing donor data including donor HCV RNA limit the ability to draw more firm conclusions from this study [13]. Additionally, a single-center retrospective study on 102 kidney transplant recipients compared late-initiation G/P for a shortened course of 8 weeks versus late-initiation sofosbuvir/velpatasvir (SOF/VEL) for 12 weeks [14]. Time to DAA initiation was again around 4 weeks. There was no significant difference in the rates of SVR12, adverse events, or complications between the two groups, suggesting that it might be safe to use a 4-week shortened treatment strategy even if DAA treatment is postponed beyond 7 days post-transplant [14]. Both studies had a short follow-up time (6 and 10 months) and were non-randomized. Data from larger multicenter randomized trials exploring early versus late DAA treatment initiation with a longer follow-up are necessary to evaluate the safest treatment strategy. Currently, the threat of potential long-term HCV-related complications makes the option of late initiation of DAA treatment as a general rule less attractive.

**Table 1:** DAA treatment in kidney transplantation from HCV RNA-positive donor to HCV-negative recipient.

Reference and Year of Study	Type of Study	*n*	Time to DAA Treatment Initiation Post-Transplant	DAA Regimen	Duration of DAA Treatment	SVR12	Adverse Events
Goldberg (2017) [9]	Open-label, single-group, pilot trial	10	3 days	Elbasvir–grazoprevir	12 weeks	100%	None
Durand (2018) [15]	Open-label, non-randomized trial	10	Immediately prior to transplantation	Elbasvir/grazoprevir, sofosbuvir added for HCV genotype 2 or 3	12 weeks	100%	None
Friebus-Kardash (2019) [16]	Retrospective study	7	5 to 37 days	Sofosbuvir-based treatment	8 to 12 weeks	100%	Arterial hypertension, loss of appetite, dizziness, sleep disorder, paresthesia
Sise (2020) [8]	Prospective, multicenter, non-randomized trial	30	2 to 5 days	Glecaprevir/pibentasvir	8 weeks	100%	None
Gordon (2023) [17]	Meta-analysis	557	0 to 76 days	Different regimens	8 days to 12 weeks	97.7%	3 cases of fibrosing cholestatic hepatitis
Feng (2022) [18]	Meta-analysis	454	0 to 76 days	Different regimens	4 to 16 weeks	100%	None
Aleyadeh (2024) [13]	Retrospective–prospective observational multicenter study comparing early and late treatment initiation regimen	158	0 to 114 days	Sofosbuvir/velpatasvir, glecaprevir/pibrentasvir (+ezetimibe), ledipasvir/sofosbuvir, elbasvir/grazoprevir	7 days to 13 weeks	100% in the early- and 94.9% in the late-treatment group	Fibrosing cholestatic hepatitis in 1 patient
Papanikolla (2024) [14]	Retrospective study	102	4 weeks	Sofosbuvir/velpatasvir, glecaprevir/pibrentasvir	8 to 12 weeks	1 patient achieved SVR only after additional therapy; all other patients achieved SVR12	None

DAA treatment, direct-acting antiviral treatment; SVR12, sustained virologic response 12 weeks after termination of treatment; HCV, hepatitis C virus.

In our case, DAA therapy was initiated prophylactically before transplantation and continued for 12 weeks, resulting in permanent absence of HCV RNA detection with no known complications. G/P and sofosbuvir were used simultaneously. Treatment failure is rare with the currently available pan-genotypic DAAs, even in patients with chronic kidney disease (CKD) and kidney transplant recipients [19]. The fixed combination of the NS3 protease inhibitor glecaprevir and the NS5A inhibitor pibrentasvir (G/P) is recommended for patients with all stages of renal insufficiency [19,20]. In patients with genotype 3 infection who are considered a more difficult-to-treat population, intent-to-treat (ITT) and modified ITT SVR12 rates were 95% and 97%, respectively [21]. However, there are limited data on the efficacy of G/P in treating uncommon HCV subtypes that are inherently resistant to NS5A inhibitors, although pibrentasvir has a higher barrier to resistance than all other NS5A inhibitors. Considering that in our case of genotype 3a HCV infection, HCV genome sequencing was unfortunately not performed to identify resistance-associated substitutions (RASs) prior to treatment initiation, the triple combination of G/P and sofosbuvir, which has also been shown to be safe and effective in patients with severe CKD, served as an alternative to treat possible complex NS5A RAS patterns [20].

The combination of G/P with tacrolimus has been shown to induce a reversible change in tacrolimus metabolism, increasing its trough levels without affecting G/P exposure. Tacrolimus dose reduction can be considered at the time of G/P initiation [22]. A recent study of interactions between G/P and tacrolimus showed that most patients had supratherapeutic levels of tacrolimus in the first two weeks after starting G/P treatment, with tacrolimus levels significantly lower after completion of G/P treatment than during treatment [14]. In the case of prophylactic/pre-emptive G/P treatment, careful monitoring of tacrolimus trough levels should be considered, especially after completion of G/P treatment. In contrast, no change in tacrolimus dose is required with sofosbuvir [23]. In our patient, we closely monitored tacrolimus trough levels at the time of initiation and discontinuation of DAAs, and no significant dose adjustment was required. Similar findings were reported by Friebus-Kardash et al. [16].

Apart from drug–drug interactions, treatment with DAAs has many potential adverse events, most of them similar to those of other medications used in transplant patients. Two months after transplantation, our patient developed neutropenia, which may be related to DAA treatment but was more likely caused by everolimus or valganciclovir treatment and resolved after discontinuation of these two drugs without the need to adjust or discontinue DAA treatment. In addition, there are reports of higher-than-expected CMV and BK viremia in HCV RNA D+/R− transplants [24]. It has been suggested that the immune system is less able to control BK virus replication in the case of HCV infection [8]. In a retrospective matched cohort study, HCV RNA D+/R− kidney transplantation was significantly associated with BK viremia ≥10,000 copies/mL or BK nephropathy, but not with BK viremia ≥1000 copies/mL. BK viremia was not associated with late DAA initiation [25]. The study suggested that HCV infection does not lead to a higher risk of BK viremia, but when infection occurs, the recipient’s immune system is less able to control BK virus replication [25]. The HCV core protein has been associated with impaired effector functions and the survival of CD8+ T cells, which play an important role in the clearance of BK viremia [26]. However, the higher incidence of severe BK viremia was not confirmed in a study by Daloul et al., which found no difference between BK viremia in HCV RNA-positive and HCV RNA-negative kidney transplant recipients. The same was shown for CMV infection and disease [27]. BK viremia is usually the result of a cumulative dose of immunosuppression, as was probably the case in our patient who no longer had HCV viremia from day 2 post-transplant and HCV probably contributed less to BK virus infection.

Moreover, Molnar et al. reported a higher than normal incidence of de novo DSAs in HCV RNA D+/R− transplantation [24]. In contrast, Gupta et al. showed that the incidence of de novo DSAs and acute cellular rejection did not exceed those observed in HCV RNA-negative kidney donors [28]. In addition, the previously mentioned meta-analysis by Gordon et al. concluded that there is insufficient evidence to compare the rates of acute rejection with HCV RNA D-/R− kidney transplantation [17]. In our patient, subclinical rejection was diagnosed 1 year after transplantation by protocol biopsy and treated with pulsed steroids and an increase in the dose of mycophenolic acid. The occurrence of rejection was probably the result of the reduction in immunosuppression when BK viremia occurred, which is common even in kidney transplants with HCV RNA-negative donors.

At the last follow-up, 19 months after transplantation, our patient was HCV aviremic and in excellent condition with stable kidney function. A favorable one-year follow-up has already been reported [29,30,31,32,33], while data on longer follow-ups are scarce. The Kidney Disease: Improving Global Outcomes (KDIGO) 2022 guidelines recommend that kidneys from HCV-infected donors should be used regardless of the HCV status of the recipient (level of recommendation 1C) but point out that there is a lack of studies on longer follow-ups, which limits the use of HCV RNA-positive kidneys [34]. It is therefore important to select recipients of HCV RNA-positive kidneys appropriately. As the literature suggests, patients with long anticipated waiting times should be considered for HCV RNA D+/R− transplantations. For these patients, remaining on the waiting list poses a greater risk than infection with HCV, which can be successfully treated in most cases [35]. Similar conclusions were reached by a national US survey of kidney transplant programs, which found an average reduction in waiting time of ≥18 months to be justification for accepting an HCV RNA-positive kidney in HCV-uninfected recipients [36]. This was also the case in our patient, who was highly sensitized. On the other hand, it should be emphasized that certain groups of HCV-negative patients should probably be excluded from offers of HCV RNA-positive donors. For example, patients with cirrhosis or a history of liver disease are generally not offered organs from HCV RNA-positive donors, as this may pose an increased risk of complications in the event of HCV transmission or resistant infection. A previous HCV infection of the recipient or a history of DAA treatment also precludes the transplantation of an HCV RNA-positive kidney into a negative recipient [36]. The (potential) need for medications with significant interactions with DAA therapy such as antiepileptic drugs, antiarrhythmic amiodarone, or high-dose proton pump inhibitors are an additional consideration before deciding on HCV RNA D+/R− transplantation [37]. In some countries, another important issue might be access to DAA treatment and insurance approval of DAA therapy for the indication of HCV RNA D+/R− transplantation, which is not the case in Slovenia. Recipients who cannot be guaranteed adequate DAA therapy in a timely manner should not be offered organs from HCV RNA-positive donors [37].

Another important factor for the risk of HCV transmission and the selection of suitable recipients for HCV RNA-positive kidneys is the donor’s HCV viral load. In a Canadian single-center study that included 30 organ transplants (10 of them were kidney transplants), the detection of viremia in the recipients was strongly associated with the donors’ HCV RNA level. DAA treatment was prophylactic but short (7 days of G/P plus ezetimibe). Recipients who never developed detectable HCV viremia had a significantly lower median donor viral load of 3.71 log10 IU/mL (range 1.18–5.46, IQR 2.25–4.21) compared to recipients that developed HCV viremia post-transplant [38]. Additionally, HCV is detected in most histologic samples of kidney tissue from HCV RNA-positive donors, whereas it is not present in kidneys from HCV-seropositive RNA-negative donors [39]. The higher the HCV viral load in plasma, the more likely it is that the virus is present in kidney tissue and in greater quantities [39]. The plasma HCV viral load can be used to predict the viral load in the kidneys. Similar findings were reported by Franco et al., who found that HCV was not present in kidney tissue from donors with plasma HCV RNA below 500,000 IU/mL [40]. Given the high donor plasma viral load in our kidney transplant recipient, we expected him to develop HCV viremia and began prompt DAA treatment. Despite the high viral load of the donor, viremia was detectable but not quantifiable in our patient only on the first day post-transplant, which is in contrast to the results of Feld et al. Regardless of the donor’s viral load HCV RNA was negative in all patients 12 weeks after transplantation in the previously mentioned study by Feld et al. [38], which is similar to the results of Franco et al. [40] and speaks in favor of an early introduction of DAA therapy. Donor plasma HCV viral load is therefore most important in cases where DAA treatment would be delayed, refused, or discontinued after transplantation.

It seems reasonable that the decision on recipient selection for HCV RNA-positive kidney transplantation should be made on a case-by-case basis, with the recipient receiving all relevant information about the risks and complications of HCV RNA D+/R− kidney transplantation, including the need for DAA treatment and its complications. Informed consent should only be obtained once the recipient has been fully informed. An important process in such cases is the shared decision making between the patient and the transplant specialist.

We are currently waiting for more studies on HCV RNA D+/R− kidney transplantation with longer follow-ups in order to be able to recommend this procedure to more patients. In a study by Franco et al. a 3-year graft and patient survival was reported to be 93.3% (*n* = 15) [40]. A post-transplant analysis of 45 patients from the THINKER and EXPANDER study showed similar results. Graft function was excellent, with a median eGFR of 65.8 mL/min per 1.73 m^2^ (IQR 56–81.5) at the three-year follow-up. In the analysis, only one patient experienced acute cellular rejection 13 months post-transplant but the overall number of patients undergoing a kidney graft biopsy was low, reported at 18% (*n* = 8), suggesting that subclinical rejection might have been missed as protocol biopsies were routinely not performed at either study center [41]. Our case showing the occurrence of subclinical rejection on protocol biopsy emphasizes the need for protocol biopsies in HCV RNA-positive kidneys. Moreover, a large retrospective multicenter study of 45,827 deceased donors, including 2551 HCV RNA-positive donors, and 75,905 kidney transplant recipients found no significant difference in the mean 5-year allograft survival rate between recipients of kidneys from HCV RNA-positive and HCV RNA-negative donors [42].

The main limitations of our case presentation are the relatively short follow-up period of 19 months after transplantation and the lack of data on HCV viral load in renal tissue, as this was not tested in our donor at the time of the zero-day biopsy. Furthermore, in addition to HCV genotyping, a genome sequencing to detect possible RASs was not performed prior to the introduction of DAAs to make a targeted choice of specific DAAs and reassure the best outcome of prophylactic HCV treatment. However, the results of this test would likely have no direct clinical impact on our patient.

Given the current data, each transplant center should decide on the selection of candidates for HCV RNA D+/R− kidney transplantation on a case-by-case basis, carefully considering the availability and choice of DAA treatment, liver function testing prior to combination DAA treatment initiation, immunosuppressive medication, and post-transplant follow-up including virologic response to DAA treatment, surveillance of rejection, and detection of opportunistic infections. Screening for possible complications such as resistance to treatment, drug interactions, or a potentially higher risk of opportunistic infections after transplantation should be performed on a regular basis.

## 4. Conclusions

HCV RNA D+/R− kidney transplantation is a feasible option even for highly sensitized recipients and enables the expansion of the organ donor pool in the search for an immunologically suitable organ. It should be reserved for selected recipients, considering the recipient’s medical history, concomitant medication, and willingness to adhere to DAA treatment. Each transplant center should have a protocol for HCV RNA D+/R− kidney transplantation that is established in collaboration with infectious disease specialists, pharmacologists, and in some countries, medical insurance companies to ensure appropriate and timely DAA treatment with minimal complications. Further studies with long-term follow-ups are needed to confirm the long-term safety of this procedure.

## Data Availability

The original contributions presented in this study are included in the article. Further inquiries can be directed to the corresponding author.

## Abbreviations

The following abbreviations are used in this manuscript:

AASLD-IDSA	American Association for the Study of Liver Diseases-Infectious Diseases Society of America
CMV	cytomegalovirus
D+/R−	positive donor to negative recipient
DAA	direct-acting antiviral
DSA	donor specific antibody
EBV	Epstein–Barr virus
G/P	glecaprevir/pibrentasvir
HCV	hepatitis C virus
HHV-6	human herpesvirus 6
ITT	intent to treat
HLA	human leukocyte antigen
IU	international unit
KDIGO	Kidney Disease: Improving Global Outcomes
MFI	mean fluorescence intensity
OPTN	Organ Procurement and Transplantation Network
PCR	polymerase chain reaction
RAS	resistance-associated substitution
RNA	ribonucleic acid
SCr	serum creatinine
SOF/VEL	sofosbuvir/velpatasvir
SVR	sustained virologic response
vPRA	virtual panel-reactive antibody
